# Resting CD4^+ ^effector memory T cells are precursors of bystander-activated effectors: a surrogate model of rheumatoid arthritis synovial T-cell function

**DOI:** 10.1186/ar2390

**Published:** 2008-03-19

**Authors:** Fionula M Brennan, Nicola MG Smith, Sally Owen, Ching Li, Parisa Amjadi, Patricia Green, Anna Andersson, Andrew C Palfreeman, Philippa Hillyer, Andrew Foey, Jonathan T Beech, Marc Feldmann

**Affiliations:** 1 Kennedy Institute of Rheumatology Division, Imperial College Faculty of Medicine, Aspenlea Road, London, W6 8LH, UK; 2 School of Biological Sciences, University of Plymouth, Drake Circus, Plymouth, PL4 8AA, UK

## Abstract

**Background:**

Previously we described a system whereby human peripheral blood T cells stimulated for 8 days in a cytokine cocktail acquired effector function for contact-dependent induction of proinflammatory cytokines from monocytes. We termed these cells cytokine-activated (Tck) cells and found that the signalling pathways elicited in the responding monocytes were identical whether they were placed in contact with Tck cells or with T cells isolated from rheumatoid arthritis (RA) synovial tissue.

**Methods:**

Here, using magnetic beads and fluorescence-activated cell sorting, we extensively phenotype the Tck effector cells and conclude that effector function resides within the CD4^+^CD45RO^+^, CCR7^-^, CD49d^high ^population, and that these cells are derived from the effector memory CD4^+ ^T cells in resting blood.

**Results:**

After stimulation in culture, these cells produce a wide range of T-cell cytokines, undergo proliferation and differentiate to acquire an extensively activated phenotype resembling RA synovial T cells. Blocking antibodies against CD69, CD18, or CD49d resulted in a reduction of tumour necrosis factor-α production from monocytes stimulated with CD4^+^CD45RO^+ ^Tck cells in the co-culture assay. Moreover, blockade of these ligands also resulted in inhibition of spontaneous tumour necrosis factor-α production in RA synovial mononuclear cell cultures.

**Conclusion:**

Taken together, these data strengthen our understanding of T-cell effector function, highlight the multiple involvement of different cell surface ligands in cell-cell contact and, provide novel insights into the pathogenesis of inflammatory RA disease.

## Introduction

Rheumatoid arthritis (RA) is a chronic inflammatory disease brought about by the activities of cells and inflammatory cytokines in the inflamed synovial membrane (for review [1]). Cells found in diseased synovium are predominantly blood derived, with large numbers of activated T cells and macrophages, fewer plasma cells and dendritic cells, together with expanded numbers of resident fibroblasts and endothelial cells [2]. Of the inflammatory cytokines, tumour necrosis factor (TNF)-α [3] was demonstrated by us to be of pivotal importance in regulating the cytokine cascade in this tissue [2]. These and other studies led to the hypothesis that TNF-α was a therapeutic candidate in RA [4], and the demonstration that TNF-α blockade in murine arthritis ameliorated disease [5] led to the testing of anti-TNF biologicals in humans [6-10]. Anti-TNF biologicals are now licensed for use in RA and well over one million people worldwide have been treated with them. Patients with other chronic inflammatory diseases have also been treated with these drugs (for review [11]). Despite the success of these anti-TNF biologicals, which protect joints from destruction [12], they are not a cure, and blockade of TNF-α has the potential to compromise the immune response (for review [13]).

In contrast to the clearly defined role of macrophage-derived cytokines in the pathogenesis of RA, the relevance and contribution of the T cells is not clear and has been challenged [14]. In particular, the expectation that the increased T cells in the synovium are a result of clonal expansion to a given antigen has not been established. An human leucocyte antigen (HLA)-restricted T-cell response to antigen is suggested, because more than 80% of Caucasian RA sufferers have a shared epitope conserved across the HLA-DR1 and HLA-DR4 haplotypes (0101, 0401, 0404 and 1402) [15]. However, no overall consensus has been reached on the potential autoantigens involved. T-cell responses to collagen type II, heat shock proteins and microbial antigens have been reported in a small proportion of RA patients (for review [16]) and, more recently, autoantibodies to deiminated citrullinated peptides have been described, suggesting that they may be important autoantigens in this disease. This aside, the concordance for disease in identical twins is still less than 15%, suggesting that other factors are of major importance.

More recently, we explored the mechanism(s) underlying chronic production of TNF-α in RA synovium and found that the spontaneous TNF-α production from macrophages is dependent upon direct interaction with T cells found in this tissue [17]. Several publications, principally from the Dayer group, describe the ability of activated T cells to induce inflammatory mediator production from monocytes in a contact-dependent manner [18-21]. T cells found in the synovial joint tissue of RA patients are predominantly memory CD4^+ ^cells [22], with unusual characteristics in that they are small, noncycling and nonapoptosing, but have features of activation [23]. We hypothesized that these T cells are stimulated in the joint not as a consequence of antigen exposure but through bystander cytokine-driven activation. To test our hypothesis, we developed a surrogate model for RA T cells by stimulating resting normal human peripheral blood lymphocytes with a cocktail of cytokines (IL-2, IL-6 and TNF-α) for 8 days, as was previously reported [24]. When fixed (to exclude mediators released by the T cells) and placed in co-culture with monocytes, proinflammatory cytokines and chemokines were produced in a cell contact-dependent manner [25,26]. Comparison of cytokine-activated T (Tck) cells with conventional T-cell receptor stimulated T (Ttcr) cells activated by immobilized anti-CD3 antibodies revealed the latter to be more activated and stimulate monocytes to produce both IL-10 and TNF-α [27]. In contrast, Tck cells induced only TNF-α and not IL-10.

We postulated therefore that Tck cells are important in the perpetuation of rheumatoid disease [25] because they induce an unbalanced, proinflammatory cytokine response from monocytes, and could thus form part of a vicious cycle. Furthermore, we demonstrated that Tck cells mimicked RA T cells in their effector function, because identical signalling pathways activated within the monocyte lead to TNF-α production [17]. The monocyte phosphoinositide 3-kinase pathway and the transcription factor NFκB were utilised in a similar manner for induction of TNFα but was different from those induced by the Ttcr cells [17]. More recently [26], we demonstrated that contact-dependent induction of seven different chemokines in monocytes was also identical between Tck and RA T cells in the utilization of nuclear factor-κB in the monocyte signalling pathway but different to that by co-culture with Ttcr cells.

In this paper we investigate the phenotype of the day 8 Tck effector cells and, by using cell sorting methodologies, we determine the phenotype of Tck cells and their precursors in resting blood. Our results demonstrate that the most potent Tck effectors are from a starting population of CD4^+^CD45RO^+ ^memory T cells. Furthermore, in culture these cells divide, become activated and express increased levels of activation, adhesion and integrin molecules. Comparison with T cells extracted from the RA joint indicate that day 8 Tck cells resemble RA T cells not only in their contact-dependent effector function but also in their active phenotype, with selective upregulation of adhesion/integrin molecules (including very late antigen [VLA]-4) that facilitate the extravasation of T cells into inflamed tissues, including RA synovium.

## Materials and methods

### Reagents

IL-6 was kindly gifted by Novartis (Vienna, Austria), IL-2 from the US National Institutes of Health, IL-10 from Schering Plough (Kenilworth, NJ, USA), TNF-α from Boehringer Ingelheim (Biberach, Germany) and IFN-γ from Bender (Wien, Austria). Lipopolysaccharide (*Salmonella typhimurium*) was purchased from Sigma (Gillingham, UK) and carboxyfluoroscein succinimidyl ester (CFSE) from Molecular Probes (Leiden, The Netherlands). Flow cytometry antibodies were purchased from BD Pharmingen (Erembodegem, Belgium). Blocking antibodies against CD18 (clone TS1/18) and CD11a (clone HI111) were from BioLegend (Cambridge, UK), anti-CD69 (clone TP1.55.3) was from Beckman Coulter (High Wycombe, Buckinghamshire, UK) and anti-CD49d (clone TA-2) was from Endogen (Rockford, Illinois, USA). Isotype control antibody (mouse IgG_1_) was purchased from BD Pharmingen. Cells cultured in RPMI/glutamine (PAA Laboratories, Pasching, Germany) were supplemented with 10% normal AB human serum (Sigma Aldrich, Dorset, UK) or 5% foetal calf serum (Biowest, Nuaillé, France), as appropriate. All reagents had under 0.1 units/ml of endotoxin, by LAL (limulus amebocyte lysate) assay (BioWhittaker, Walkersville, MD, USA).

### Isolation of lymphocyte subpopulations by magnetic beads

Buffy coats were purchased from the North London Blood Transfusion Service (Colindale, UK) and peripheral blood mononuclear cells isolated by Lymphoprep™ (Cedarlane Laboratories Ltd, Ontario, Canada) centrifugation followed by elutriation, as described previously [17]. Lymphocyte subsets were isolated using CD4 or CD8 isolation kits (Dynal, Invitrogen, Paisley, UK), in accordance with the manufacturer's instructions. CD45RO^+ ^cells were isolated negatively using memory CD4^+ ^T-cell isolation kit from Miltenyi Biotec (Guildford, Surrey, UK), in accordance with the manufacturer's instructions.

### Isolation of T cell sub-populations by cell sorting (fluorescence-activated cell sorting)

A cocktail of anti-CD4 with either anti-CD45RA or anti-CD45RO antibodies was used to sort negatively for memory or naïve populations, respectively. A cocktail of anti-CD4, anti-CD45RA and anti-CCR7 (anti-CC chemokine receptor 7) antibodies was used to sort the CD4^+ ^central (CD45RA^-^CCR7^-^) and effector memory (CD45RA^-^CCR7^+^) populations on day 0. For the day 8 sort, CCR7 antibody was used to sort positively the central and effector memory cells from the CD4^+^CD45RO^+ ^Tck population that was previously isolated at day 0 using the memory CD4^+ ^T-cell isolation kit from Miltenyi Biotec, as described above. The resulting cell purity was routinely assessed at more than 95%. Cells were sorted on a FACSVantage SE (BD Biosciences, Cambridge, UK).

### Tck generation and monocyte co-culture

Tck cells were generated from resting blood with IL-2, IL-6 and TNF-α, as previously described [17]. Supernatants were harvested and T-cell-derived cytokine (IFN-γ, granulocyte macrophage colony-stimulating factor, lymphotoxin-α and IL-10) levels were measured using sandwich ELISA (BD Pharmingen). Tck cells were co-cultured with human peripheral blood monocytes for 18 hours, as previously described [17], and TNF-α levels in supernatants were assayed by sandwich ELISA (Pharmingen, San Diego, CA).

### Isolation of RA synovial membrane mononuclear cells and phenotypic analysis

Mononuclear cells were obtained from RA synovial tissue, as previously described [17]. Tissues were from joint replacement surgery specimens provided by the Orthopedic/Plastic Surgery Department, Charing Cross Hospital, London, UK (covered by ethics approval RREC 1752), after receiving signed and informed patient consent and anonymization. Peripheral blood lymphocytes or RA synovial mononuclear cells were incubated with fluorochrome-labelled antibodies and analyzed on a Becton-Dickinson LSR I flow cytometer (BD Biosciences). A total of 5,000 synovial lymphocytes were collected and results were analyzed using CellQuest Pro software (BD Biosciences).

### CFSE assay of cell proliferation

Lymphocytes were resuspended in phosphate-buffered saline with 2.5 to 5 μmol/l CFSE at room temperature for 10 minutes and washed extensively before culture. At day 8, cells were counterstained with antibodies to cell surface markers. In all, 50,000 events were routinely collected by flow cytometry.

### Blockade of CD18, CD69, CD49d and CD11a on Tck cells and RA synovial mononuclear cells

Day 8 Tck cells were fixed in 1% paraformaldehyde at 4°C before incubation with 1.25 μg per million cells of blocking antibody against CD18, CD69, CD49d, or CD11a. Isotype control antibody was also included. After 30 minutes, Tck cells were washed to remove excess antibodies and co-cultured with fresh monocytes at a ratio of 8:1 in the presence of purified human IgG (5 μg/ml). Supernatants were collected after 18 hours and TNF-α was assayed by ELISA. RA synovial mononuclear cells (MNCs) were incubated in triplicate for 24 hours at 0.5 × 10^6^/ml in the presence or absence of 5 μg/ml of blocking antibody against CD18, CD69, CD49d, or isotype control. Supernatants were collected for TNF-α analysis by ELISA.

### Statistical analysis

Results were analysed using Prism 4 software (GraphPad Software Inc., San Diego, CA, USA) and statistical differences between groups were analyzed using Wilcoxon rank test, Student's *t*-test, or one-way analysis of variance with Bonferroni *post hoc *corrections as appropriate. Correlation association between groups of data was analysed using nonparametric (Spearman) correlation analysis.

## Results

### Phenotype of day eight Tck cells following cytokine stimulation

**Figure 1:**
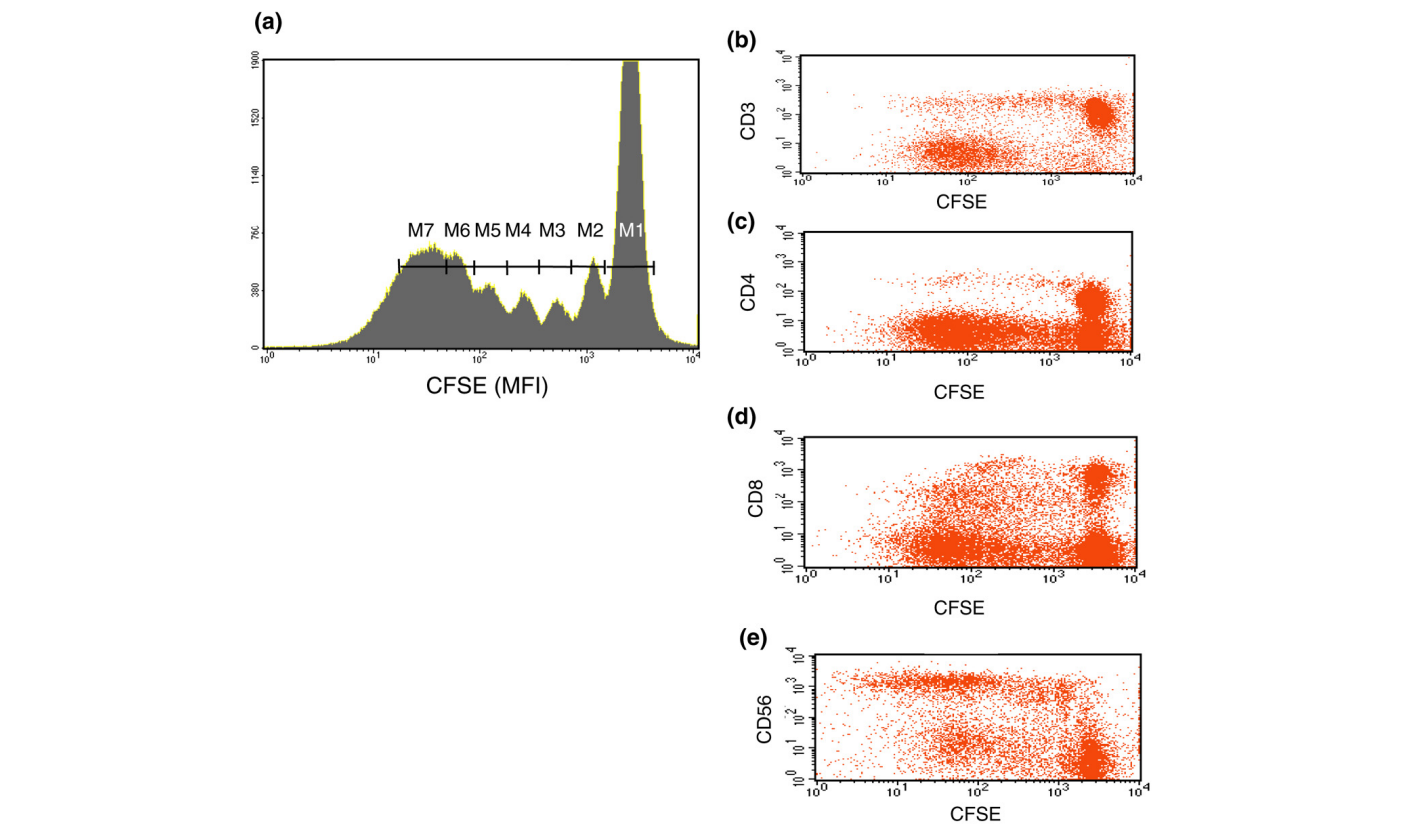
Proliferation pattern of cytokine-stimulated lymphocytes. **(a) **Resting peripheral blood lymphocytes (PBLs) undergo cell divisions when they are stimulated with cytokines over 8 days. Freshly elutriated lymphocytes were labeled with 5 μmol/l carboxyfluoroscein succinimidyl ester (CFSE) and stimulated with IL-2, IL-6 and tumour necrosis factor-α for 8 days. The number of cell divisions was analysed on day 8 using CellQuest software. Markers (M1 to M7) indicate sequential cell divisions. **(b-e) **Proliferation of subpopulations of PBLs stimulated with cytokines. CFSE-labelled PBLs were counterstained with antibodies to CD3 (panel b), CD4 (panel c), CD8 (panel d) and CD56 (panel e). On day 8 the number of cell divisions for each subpopulation of PBLs was acquired and analyzed using CellQuest software. Results for all figures are shown from one donor representative of seven different experiments.

Figure 1a illustrates the CFSE dilution of Tck cells at day 8 counterstained for CD3 (Figure 1b), CD4 (Figure 1c), CD8 (Figure 1d) and CD56 (Figure 1e). At day 8 approximately 40% of cells were undivided (peak M1), whereas 60% of cells (peaks M2 to M7) underwent up to six rounds of cell division (Figure 1a). The CD3^+^CD4^+ ^and CD3^+^CD8^+ ^cells were mostly found within the undivided population (M1; Figure 1b,c,d), whereas most CD56^+ ^natural killer (NK) cells (Figure 1e) underwent several rounds of cell division. Pooling data from seven different donors indicated that overall there was a small increase in cell number from 1 million/ml at day 0 to 1.43 million/ml at day 8, accounted for predominantly by increases in CD3^-^CD56^+ ^NK cells from 0.07 to 0.25 million/ml (about 3.5-fold) and CD3^+^CD8^+ ^T cells from 0.29 to 0.64 million/ml (2-fold). CD3^+^CD4^+ ^T cells numbers were increased less, from 0.51 to 0.67 million/ml. CD19^+ ^B cells did not survive the 8-day culture (data not shown).

### Tck effector function resides within the CD4^+ ^T-cell subset

**Figure 2:**
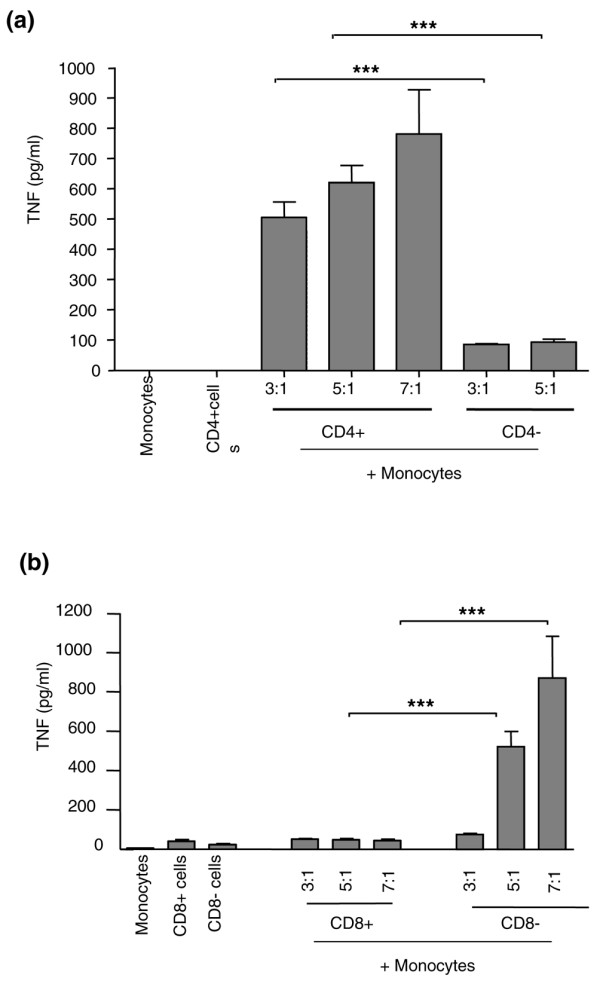
The effector function of cytokine-stimulated lymphocytes resides within the CD4^+ ^population. **(a) **Effector function of the CD4^+ ^cytokine-activated T (Tck) cell population compared with that of the CD4^- ^Tck population. Lymphocytes were positively separated using CD4^+ ^magnetic beads (Dynal) before stimulation for 8 days with IL-2, IL-6 and tumour necrosis factor (TNF)-α. On day 8 lymphocytes were co-cultured with autologous monocytes (at the indicated ratios) for 18 hours before supernatants were removed and assayed for TNF-α by ELISA. Supernatants from cultures of monocytes and T cells alone as negative controls contained under 50 pg/ml. This experiment is representative of seven different donors. ***P < 0.001 comparing CD4^+ ^versus CD4^- ^at a ratio of 3:1, or comparing CD4^+ ^versus CD4^- ^at a ratio of 5:1 using one-way analysis of variance (ANOVA) with Bonferroni's multiple comparison. Because of limited CD4^- ^cell numbers, a comparison at a ratio of 7:1 of could not be performed in this experiment. **(b) **Effector function of CD8^+ ^Tck population compared with that of CD8^- ^Tck population. Lymphocytes were positively separated using CD8^+ ^magnetic beads (Dynal) before stimulation for 8 days with IL-2, IL-6 and TNF-α. On day 8 lymphocytes were co-cultured with autologous monocytes (at the indicated ratios) for 18 hours before the supernatants were removed and assayed for TNF-α by ELISA. Supernatants from cultures of monocytes and T cells alone as negative controls contained under 50 pg/ml. This experiment is representative of seven different donors. ***P < 0.001 comparing CD8^+ ^versus CD8^- ^at a ratio of 5:1, or comparing CD8^+ ^at versus CD8^- ^at a ratio of 7:1 using one-way ANOVA with Bonferroni's multiple comparison.

Figure 2a demonstrates that effector function (induction of monocyte TNF-α in a dose-dependent manner from 500 to 800 pg/ml by day 8 Tcks) resided in cells derived at day 0 from the CD4^+ ^(*P *< 0.001) but not the CD4^- ^population (which included NK cells, CD8^+ ^cells and NK T cells). Less TNF-α (<100 pg/ml) was induced with day 8 Tck cells derived from a starting population of CD4^- ^cells (CD8^+^, NK T and NK cells). To exclude the possibility that ligation of CD4 by the anti-CD4 magnetic beads affected the result, Figure 2b shows that the CD8^+ ^cells enriched at day 0 (which included CD8^+ ^T cells and NK T cells) did not induce an effector response at day 8 (<50 pg/ml). In contrast, the CD8^- ^enriched cells (which were predominantly CD4^+ ^along with CD56^+ ^NK cells) induced a potent Tck effector response (*P *< 0.001) in a dose-dependent manner at day 8 (75 to 875 pg/ml).

### CD4^+^CD45RO^+ ^(memory) Tck cells induce greater monocyte TNF-α

**Figure 3:**
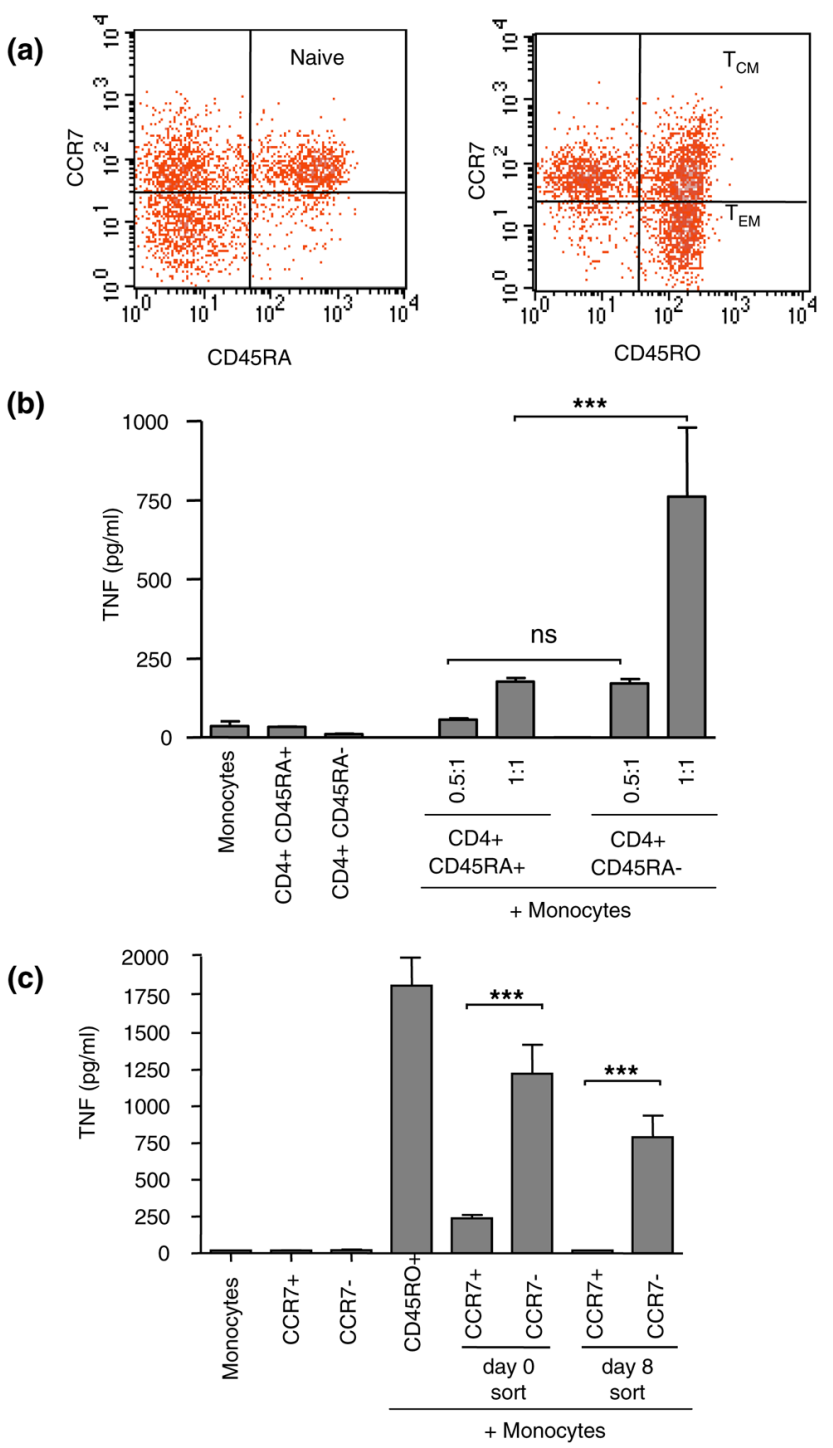
Memory T cells exhibit greater effector function than naïve T cells. **(a) **Fluorescence-activated cell sorting analysis of CD4^+ ^subpopulations. Resting CD4^+ ^lymphocytes in normal peripheral blood contains CD45RA^+^CCR7^+ ^(naïve) lymphocytes (left panel) together with CD45RO^+^CCR7^+ ^central memory (T_CM_; right panel) and CD45RO^+^CCR7^- ^effector memory populations (T_EM_; right panel). **(b) **Effector function resides mainly within the CD4^+^CD45RO^+ ^population. Lymphocytes were sorted into naïve (CD4^+^CD45RA^+^) and memory (CD4^+^CD45RO^+^) populations at time zero, before stimulation for 8 days with IL-2, IL-6 and tumour necrosis factor (TNF)-α. Lymphocytes were then co-cultured with monocytes at a ratio of 0.5:1 and 1:1 for 18 hours and the supernatants assayed in triplicate for TNF-α production by ELISA. ***P < 0.001 comparing CD4^+^CD45RA^+ ^versus CD4^+^CD45RO^+ ^populations using one-way analysis of variance (ANOVA) with Bonferroni's multiple comparison. **(c) **Effector function resides predominates within CD4^+ ^effector memory population. CD45RO^+ ^cells were further sorted by flow cytometry into CCR7^+ ^and CCR7^- ^populations at day 0 (and stimulated for 8 days with cytokines) or at day 8 (after cytokine expansion) and then co-cultured with monocytes at a ratio of 1:1 for 18 hours, and the supernatants assayed in triplicate for TNF-α production by ELISA. ***P < 0.001 comparing CD4^+^CD45RO^+^CCR7^- ^versus CD4^+^CD45RO^+^CCR7^+ ^after sorting at day 0 sort or at day 8 using one-way ANOVA with Bonferroni's multiple comparison. In panels b and c monocytes and T cells cultured alone as negative controls produced under 20 pg/ml TNF-α. Results are shown from one donor representative of five different experiments (panel b) and three different experiments (panel c). CCR, CC chemokine receptor.

CD4^+^CD45RO^+ ^(memory) T cells and CD4^+^CD45RA^+ ^(naïve) T cells [28], enriched at day 0 (Figure 3a), were sorted by flow cytometry from the same donor based on CD45RA expression and expanded for 8 days. Figure 3b demonstrates that the memory T cells when cultured at a ratio of 1:1 with monocytes induced significantly higher levels (*P *< 0.001) of TNF-α (6.7-fold more) than did the naïve T cells at the comparable ratio. This result was confirmed in five independent experiments. The most potent inducers of monocyte TNF-α production within the CD4^+ ^memory population was found to reside within the effector memory population (CD4^+^CD45RO^+^CCR7^-^), irrespective of whether the cells were sorted before *ex vivo *cytokine expansion or afterward (Figure 3c). Sixfold more TNF-α was produced by the effector memory cells (CCR7^-^) separated at day 0 (*P *< 0.001) compared with central memory cells (CCR7^+^) and about 13-fold more TNF-α produced by effector memory cells separated at day 8 (*P *< 0.001) compared with central memory cells. This result was confirmed in three independent experiments.

### Phenotype of day eight CD4^+^CD45RO^+ ^Tck cells after cytokine stimulation

Table 1 summarizes the pooled median mean fluorescence intensity (MFI) values and percentage of cells positive for cell surface molecules at days 0 and 8 on the CD4^+^CD45RO^+ ^cells from independent experiments. T-cell activation markers (CD25, CD69 and HLA-DR) were significantly increased at day 8, with median MFI increases of greater than sevenfold for CD25 (*P *< 0.0001), eightfold for CD69 (*P *< 0.0001) and threefold for HLA-DR (*P *= 0.0052), respectively. CD25^+ ^cells increased from 9% at day 0 to 18% at day 8, indicating expansion of some cells in culture. Day 8 Tck cells also exhibited a significant increase in CD69 expression (Table 1), with 27% of the cells positive for CD69 at day 8. HLA-DR was also increased but to a lesser extent, from 9% at day 0 to 15% at day 8.

**Table 1:** Expression of activation markers, adhesion molecules and chemokine receptors in CD4^+^CD45RO^+ ^Tck cells

Cell phenotype	Median MFI (range)/percentage positive (%) index		Change^a^	*P*	*n*
				
	Day 0	Day 8			
CD25	8.0 (2–49)/9%	61 (6–884)/18%	7.5	<0.0001	40
CD69	5.0 (2–43)/1.1%	41 (5–552)/26.7%	8	<0.0001	41
HLA-DR	19 (4–130)/9%	60 (11–238)/15%	3	0.0052	15
CD62L	22 (8–255)/17%	339 (7–1,040)/58%	15	<0.0001	31
CD18	107 (9–1,026)/97.5%	319 (53–2,040)/98.5%	3	0.0085	21
CD29	85 (6–668)/81%	152 (14–582)/97%	2	0.0132	35
CD49d	180 (5–634)/73.3%	465 (13–1,461)/82.3%	2.6	<0.0001	42
CCR7	12 (4–57)/7.4%	13 (3–138)/8.6%	1	NS	42
CXCR4	52 (18–494)/29%	270 (124–896)/86.4%	5	<0.0001	21
CXCR3	32.3 (9–309)/34.3%	40.4 (17–129)/34.0%	1	NS	21
CCR5	8.3 (4–28)/2%	10.7 (4–262)/6%	1	NS	21

Statistically significant differences in mean fluorescence intensity (MFI) between day 0 and day 8 values from different experiments were calculated using Wilcoxon rank test. ^a^Change index is calculated with reference to Day 0. CCR, CC chemokine receptor; CXCR, CXC chemokine receptor; HLA, human leucocyte antigen; NS, not significant; Tck, cytokine-activated T.

Adhesion marker expression was more variable. Firstly, L-selectin (CD62L), a potential marker of central memory cells was upregulated (15-fold) in the day 8 Tck cells (*P *< 0.0001). An expansion in the central memory population is not likely because CCR7^+ ^expression (a more robust marker of central memory cells) remained low in the day 8 CD4^+^CD45RO^+ ^Tck cells (Table 1). Interestingly, CD62L expression peaked at day 6, followed by a significant decrease in expression at day 8 (data not shown). This coincided with detection of soluble CD62L in the culture supernatants (>150 ng/ml by day 6), indicating shedding of CD62L (data not shown). Using nonparametric (Spearman) correlation analysis (Table 2), it was observed that CD62L expression at day 8 negatively correlated (*r *= -0.4652; *P *= 0.00084) with TNF-α production in the co-culture system (n = 31).

**Table 2:** Relationship between day 8 Tck phenotype (MFI) and effector function (TNF-α production from co-cultured monocytes)

Cell phenotype	*n*	*r *(correlation)	*P*
CD25	40	-0.2558	NS
CD69	41	0.00053	NS
HLA-DR	15	0.4094	NS
CD62L	31	-0.4652	0.00084
CD18	21	0.1442	NS
CD29	35	0.2167	NS
CD49d	42	0.3314	0.032
CCR7	42	-0.1711	NS
CXCR4	21	-0.13	NS
CXCR3	21	0.02341	NS
CCR5	21	-0.3948	NS

Day 8 expression of cell surface markers (mean fluorescence intensity [MFI]) was correlated with effector function (tumour necrosis factor [TNF]-α production from monocytes in cytokine-activated T [Tck] cell:monocyte co-culture) using nonparametric (Spearman) correlation analysis. Results are expressed as correlation values (*r*) and level of significance.

The proportions of cells positive for the adhesion markers CD18, CD29 and CD49d did not increase substantially in culture, because more than 70% of the resting cells were already positive for these markers at day 0. However, the median MFI expression did increase for each molecule, with threefold (CD18; *P *< 0.0085), twofold (CD29; *P *= 0.0132) and threefold (CD49d; *P *< 0.0001) increases at day 8. Interestingly, using nonparametric (Spearman) correlation analysis (Table 2), it was observed that VLA-4 (CD49d) expression at day 8 positively correlated (*r *= 0.3314; *P *< 0.032) with TNF-α production in the co-culture system (n = 42). It is unlikely that this ligand is binding vascular cell adhesion molecule (VCAM)-1 (its counter ligand) on monocytes because this molecule is not found on peripheral blood monocytes. However, this may indicate that the most activated cells have the potent effector function in the co-culture assay and that CD49d is a surrogate marker for this activity.

Chemokine receptor expression was variable and lower than that of the adhesion molecules. Only CXC chemokine receptor (CXCR)4 increased significantly (fivefold; *P *< 0.0001) over the 8-day culture period, with 86% cells positive at day 8 compared with 29% at day 0. Increases in CXCR3 and CCR5 expression were also observed but did not reach statistical significance. Levels of CCR7 marker for naïve (CD45RA^+^) and central memory (CD45RO^+^) cells were low (3%) and remained unchanged over the 8-day period.

### Phenotype of proliferated CD4^+^CD45RO^+ ^Tck cells following cytokine stimulation

**Figure 4:**
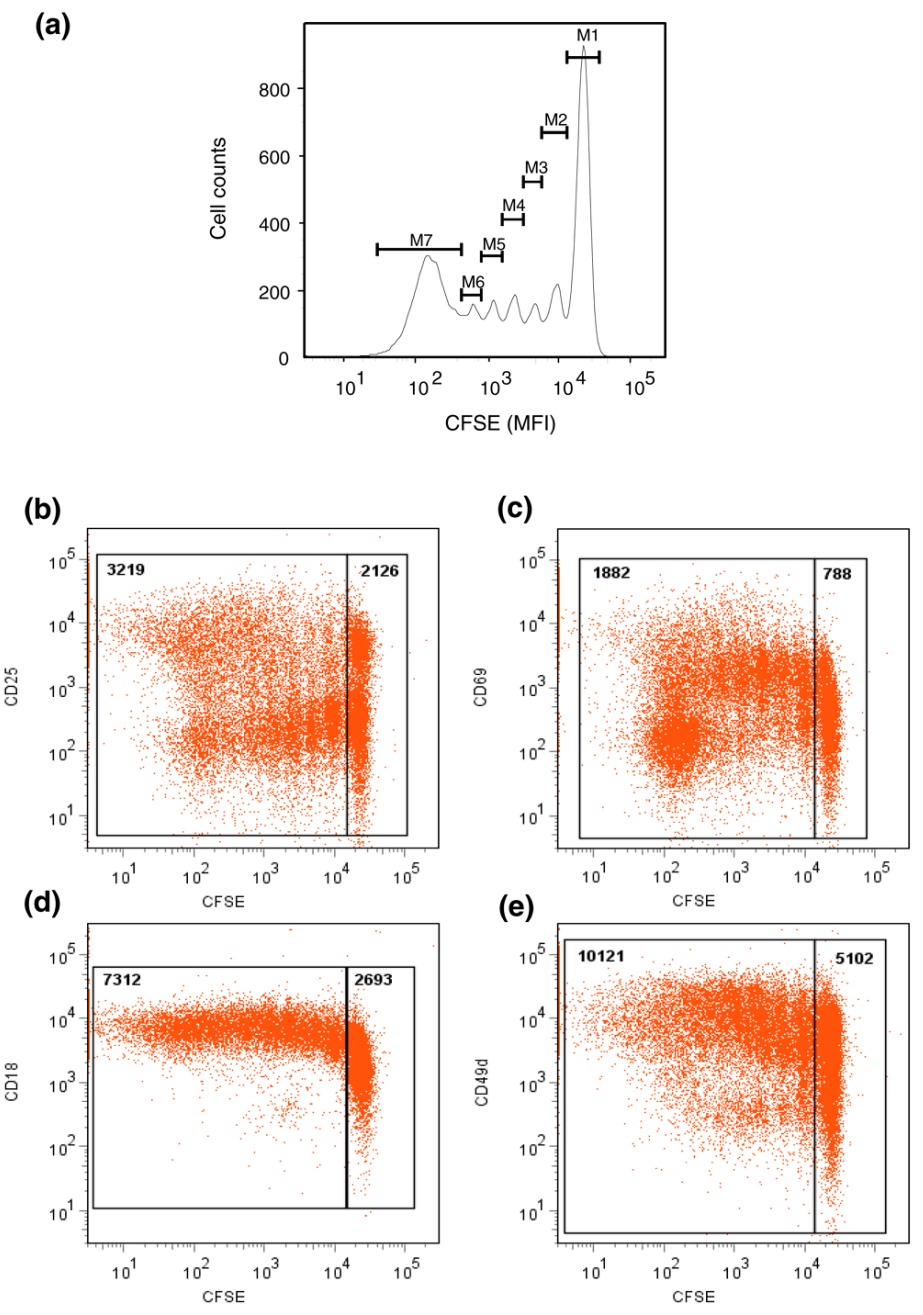
Proliferation pattern and phenotype of day 8 CD4^+^CD45RO^+ ^Tck cells after cytokine stimulation. Freshly isolated CD4^+^CD45RO^+ ^cells were labeled with 2.5 μmol/l carboxyfluoroscein succinimidyl ester and stimulated with IL-2, IL-6 and tumour necrosis factor (TNF)-α for 8 days. **(a) **The number of cell divisions was analyzed on day 8 using FlowJo 7.2.2 software (Tree Star, Inc., Oregon, USA). Cells were counterstained with antibodies against **(b) **CD25, **(c) **CD69, **(d) **CD18 and **(e) **CD49d, with mean fluorescent intensity (MFI) of these markers assessed in the undivided (M1) peak versus the divided (M2 to M7) fractions. Results are shown in the gates. Figure representative of four independent experiments. Tck cell, cytokine-activated T cell.

Figure 4a illustrates the CFSE dilution of CD4^+^CD45RO^+ ^Tck cells at day 8 (representative of four different donors) counterstained for CD25 (Figure 4b), CD69 (Figure 4c), CD18 (Figure 4d) and CD49d (Figure 4e). Over four independent donors, an average of 55% of cells were undivided (M1) whereas 45% of cells (peaks M2 to M7) underwent up to six rounds of cell divisions. The MFI of both activation markers and adhesion molecules were found to be higher in the divided population. The MFI of CD25 (Figure 4b) was 3,219 in the divided as compared with 2,126 in undivided cells; the MFI of CD69 (Figure 4c) was 1,881 compared with 788; the MFI of CD18 (Figure 4d) was 7,312 compared with 2,693; and CD49d expression (Figure 4e) was 10,121 compared with 5,102.

### Day 8 CD4^+^CD45RO^+ ^Tck cells phenotypically resemble RA synovial T cells

**Figure 5:**
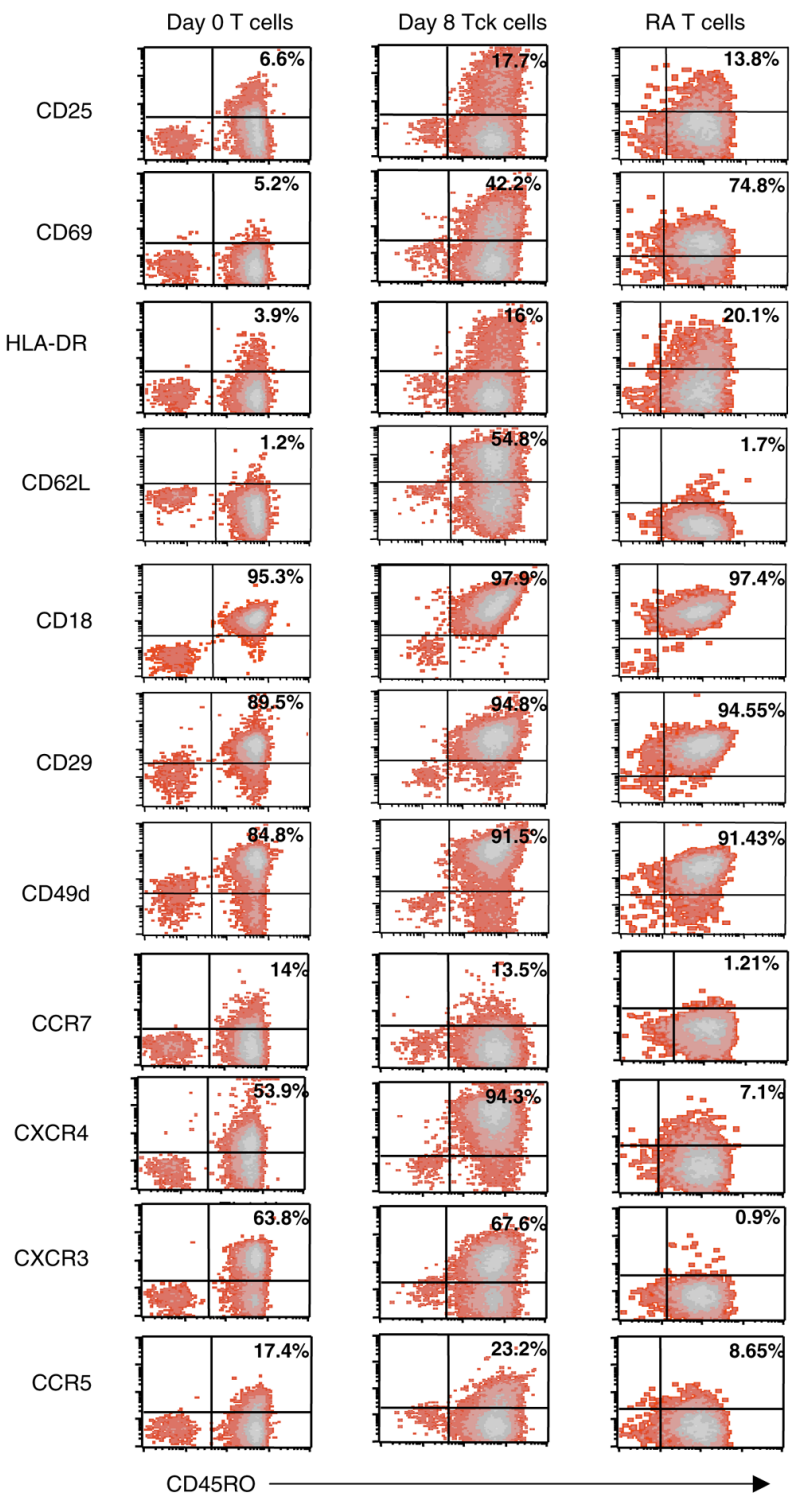
Day 8 CD4^+^CD45RO^+ ^Tck cells phenotypically resemble rheumatoid synovial T cells. Freshly elutriated peripheral blood lymphocytes (PBLs) from healthy donors were stained with antibodies against CD4 and CD45RO in combination with antibodies against CD25, CD69, HLA-DR, CD62L, CD18, CD29, CD49d, CCR7, CXCR4, CXCR3, or CCR5 at time 0 (left panels) and day 8 (middle panels) after culture with IL-2, IL-6 and tumour necrosis factor (TNF)-α (as detailed in Materials and methods). Rheumatoid synovial cells were also stained with the same panel of antibodies as PBLs. In all, 1 × 10^4 ^PBLs and 5 × 10^3 ^synovial T cells were collected and the expression of different cell markers on the CD4^+ ^gated lymphocyte population versus CD45RO^+ ^expression was analyzed on the CellQuest Pro software. CCR, CC chemokine receptor; CXCR, CXC chemokine receptor; HLA, human leucocyte antigen.

Figure 5 illustrates the phenotype from a representative experiment using peripheral blood CD4^+^CD45RO^+ ^lymphocytes at day 0 (left) and after 8 days of culture with cytokines (middle) compared with CD4^+ ^T cells within RA synovial membrane tissue (right). CD25 expression was present in a small proportion (9%) of resting lymphocytes, which was increased (18%) on day 8 Tck cells. A small proportion of RA synovial T cells in this sample were also CD25^+ ^but not to the same extent as day 8 Tck cells. The activation markers CD69 and HLA-DR were clearly elevated on day 8 Tck cells and were similarly elevated on RA synovial T cells. CD62L was absent from resting T cells but increased on the day 8 Tck cells. However, RA synovial T cells were negative for this selectin. Adhesion molecules CD18, CD29 and CD49d were present on resting T cells but further upregulated in day 8 Tck cells, with similar expression to that seen on RA synovial T cells. The chemokine receptor CCR7, a 'marker' of naïve and central memory T cells, was expressed on the day 0 resting T cells. Its expression was absent on the RA synovial T cells and day 8 Tck cells. The chemokine receptors CXCR4, CXCR3 and CCR5 were all increased on day 8 Tck cells to varying extents, but in the RA synovial tissue examined here only CXCR3 was found to be upregulated on the RA synovial T cells. However, on the other RA synovial specimens examined (data not shown) both CXCR4 and CCR5 expression was abundant, which is consistent with numerous published reports (for review [29]).

### Day 8 CD4^+^CD45RO^+ ^Tck effector function is blocked with neutralizing antibodies to CD69, CD18 and CD49d

**Figure 6:**
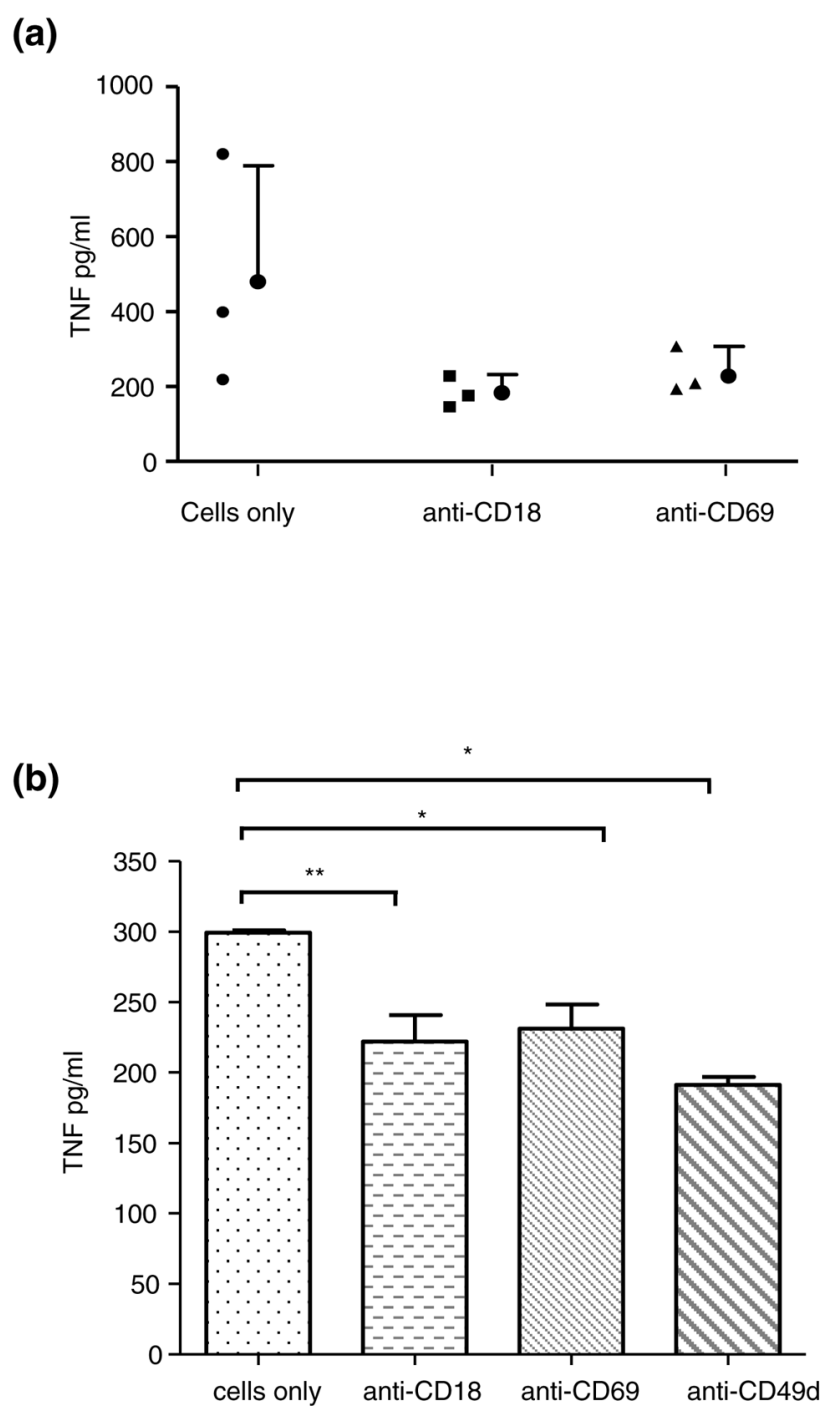
CD69, CD18 and CD49d blockade resulted in reduced TNF-α in Tck co-cultures and rheumatoid synovium culture. **(a) **CD4^+^CD45RO^+ ^cells were fixed after 8 days of cytokine stimulation with 1% paraformaldehyde and then co-incubated with blocking antibodies against CD18 and CD69, as described in Materials and methods. Cytokine-activated T (Tck) cells were co-cultured with fresh monocytes at a ratio of 8:1. Supernatants were harvested after 18 hours and TNF-a production measured by ELISA. This figure represents three experiments conducted in different donors. **(b) **Rheumatoid arthritis (RA) synovial cell mononuclear cells from RA donor 2 (see Table 3) were cultured in triplicate in the presence of antibodies against CD69, CD18, or CD49d for 24 hours, as described in Materials and methods. Supernatants were harvested and spontaneous TNF-α production was measured using ELISA. **P < 0.01 and *P < 0.05, as assessed by one-way analysis of variance with *post hoc *Bonferroni correction.

Having demonstrated that day 8 CD4^+^CD45RO^+ ^Tck cells exhibit a differentiated phenotype, we next determined whether blocking these surface molecules with neutralizing antibodies would result in inhibition of the effector response. Thus, in a series of experiments CD4^+^CD45RO^+ ^Tck cells were pre-incubated with neutralizing antibodies to CD69, CD18, CD49d, CD11a, or isotype control antibodies (as described in Materials and methods, above), and TNF-α production from monocytes was assessed in the co-culture assay. In three independent experiments (Figure 6a), blockade of CD69 resulted in an overall inhibition of 37% (*P *< 0.05), with TNF-α levels reduced from 479.6 ± 310.3 pg/ml to 227.7 ± 79.6 pg/ml. Blockade of CD18 resulted in an overall inhibition of 50% (*P *< 0.01) with TNF-α levels reduced from 479.6 ± 310.3 pg/ml to 183.8 ± 48.5 pg/ml. Blockade of CD49d resulted in 70% inhibition of TNF-α production from 69 ± 24 pg/ml to 10 ± 5 pg/ml (*P *= 0.04) in one experiment, although in a further three experiments no inhibition was observed (data not shown). Blockade of CD11a had no effect (data not shown).

### Spontaneous production of TNF-α and IL-6 is blocked in RA synovial MNC cultures with neutralizing antibodies to CD69, CD18, or CD49d

Having demonstrated that day 8 CD4^+^CD45RO^+ ^Tck cells phenotypically resemble RA synovial T cells, we next explored whether ligation events involving CD69, CD18, or VLA-4 (CD49d) were instrumental in inducing/maintaining proinflammatory cytokine production in RA synovial MNC *ex vivo *cultures. Thus, in a series of experiments on different RA donors, synovial MNC cells were incubated in triplicate in the presence or absence of neutralizing antibodies to CD69, CD18, CD49d, or an isotype control. Table 3 illustrates the percentage inhibition of spontaneous TNF-α production from four different RA synovial MNC cultures compared with cells only control (similar levels were obtained with isotype antibody control). Blockade of CD69 had a modest (approximately 21%) inhibitory effect upon TNF-α production in all RA synovial cultures, although in one of these donors this change was not significant (Table 3). Blockade of CD18 resulted in TNF-α inhibition in three out of the four donor RA cultures, with as much as 50% inhibition observed in donor 4. Blockade of CD49d resulted in inhibition of TNF-α in only two out of the four RA donor cultures. In RA donor two (Figure 6b), blockade of CD69, CD18, or CD49d all resulted in significant inhibition TNF-α production (data illustrated as mean TNF-α production [pg/ml] from triplicate cultures).

**Table 3:** Modulatory effect on spontaneous TNF-α production following blockade of cell surface ligands in RA MNC cultures

RA donor	Anti-CD69		Anti-CD18		Anti-CD49d	
	Inhibition (%)	*P*	Inhibition (%)	*P*	Inhibition (%)	*P*
1	17	<0.01	0	NS	0	NS
2	23	<0.05	22	<0.01	36	<0.01
3	22	NS	26	NS	23	NS
4	ND	NS	51	<0.01	0	NS

Rheumatoid arthritis (RA) synovial mononuclear cell (MNC) cells were cultured in triplicate with anti-CD69, anti-CD18, or anti-CD49d antibodies at a final concentration of 5 μg/ml for 24 hours at 37°C, as described in Materials and methods. Spontaneous tumour necrosis factor (TNF)-α production was measured in the supernatants by ELISA and results expressed as percentage inhibition relative to the control wells (cells only). Significance (*P *value) was calculated using one-way analysis of variance with *post hoc *Bonferroni correction.

### Cytokine production by CD4^+^CD45RO^+ ^Tck cells in culture

Supernatants from the day 8 culture CD4^+^CD45RO^+ ^Tck cells were screened for T-cell cytokines and significant amounts of lymphotoxin-α (median 358 pg/ml), IFN-γ (median 534 pg/ml) and GM-CSF (median 2,095 pg/ml) were found, with lesser amounts of IL-10 (median 30 pg/ml; Table 4). IL-17 was not detected in the Tck culture supernatants at day 8 but was induced if at day 5 the Tck cell cultures were treated with 2 ng/ml recombinant IL-23 (data not shown).

**Table 4:** Cytokine levels found in day 8 CD4^+^CD45RO^+ ^Tck cell culture supernatants

Cytokine	*n*	Median levels (pg/ml)	Range (pg/ml)
Lymphotoxin-α	25	358	(38–7,306)
IFN-γ	32	534	(8–21,280)
GM-CSF	21	2,095	(603–8,043)
IL-10	36	30	(0–310)

Supernatants from day 8 cytokine-activated T (Tck) cell cultures were harvested and cytokines detected by ELISA, as described in Materials and methods. GM-CSF, granulocyte monocyte colony-stimulating factor.

## Discussion

Although TNF-α is now acknowledged to be important in the pathology of RA [11], the mechanisms that lead to its dysregulated production remain unknown but are of importance to the development of improved therapies. Several pieces of evidence from our group and others have emerged to indicate that macrophage-derived TNF-α in inflamed synovial tissue is T cell and contact dependent [17,21,30-32]. In order to study this mechanism in more detail, we developed a human surrogate model system based on cytokine activation of peripheral blood lymphocytes, and found that after 8 days these cytokine-activated T cells (but not those activated by crosslinked anti-CD3) mimicked the effector function of rheumatoid T cells [17,25]. Importantly, the differences between Tck cells and those derived from antigen activation provided an opportunity for selective inhibition of pathogenic T cells. In this report we characterize the generation of day 8 Tck cells in terms of the induced lymphocyte proliferation, cell surface phenotype and cytokine production. Furthermore, by a series of cell sorting experiments, we not only identify the phenotype of the most potent effector cell at day 8 but also, based upon this knowledge, uncover the precursor phenotype of Tck cells within normal resting peripheral blood lymphocyte population. This may have implications for our understanding of the generation of pathogenic T cells in the highly proinflammatory environment of the rheumatoid synovium.

Analysis of the lymphocytes expanded from human peripheral blood with the cytokine cocktail in our day 8 Tck cultures revealed a significant expansion in CD3^-^CD56^+ ^cells (NK population), with a lower overall change in the number of CD3^+^CD56^- ^T cells over the 8 days, despite cell division within the CD8^+ ^population. The lack of change in total cell number at day 8 is apparently due to apoptosis in approximately 30% of the CD3^+ ^cells (data not shown), most likely because of depletion of growth factors in these cultures, particularly when expanded together with the rapidly proliferating NK cells. Indeed, if the human peripheral blood lymphocytes were expanded with IL-15 alone (previously to generate effective Tcks cells [25]), the expansion of NK cells at day 8 was even more marked. Furthermore, if the NK cells were separated at day 8 from the CD3^+ ^T cells, they also exhibited contact effector function on monocytes but in a phosphoinositide 3-kinase dependent manner (unpublished data), similar to that induced by anti-CD3 antibody stimulated peripheral blood lymphocytes [17,26,33]. Only the CD3^+ ^cells within the day 8 Tck cells (and not the CD3^-^CD56^+^) exhibited effector function identical to that of RA synovial T cells, and thus we focused our attention on these cells.

Closer analysis and enrichment of the CD3^+ ^subset phenotype (at either day 0 or day 8) revealed that the most efficient effector cell resided within the CD3^+^CD4^+^CD45RO^+ ^memory subset, and more specifically within the effector memory population (CCR7^-^). The day 8 Tck cells resembled RA synovial T cells, particularly in terms of the increased expression of several adhesion molecules including VLA-4. The latter is of particular interest because, based on its overall expression at day 8, it was the only ligand to correlate positively with the ability to induce monocyte production of TNF-α (Table 2). Synovial T cells express high levels of both the alpha (CD49d) and beta (CD29) chains of VLA-4 and exhibit *ex vivo *an enhanced ability to bind to fibronectin fragments (a VLA-4 ligand) [34]. Furthermore, an alternate VLA-4 ligand, namely VCAM-1, is expressed selectively on RA endothelium and macrophages in the lining layer of the synovium, and is inhibited following anti-TNF-α therapy in the clinic [35]. It has been hypothesized that VLA-4 facilitates extravasation of lymphocytes across endothelium (via VCAM-1) and their retention in the joint tissue (via fibronectin binding and/or VCAM-1). To investigate this further, we intend to explore the ability of VLA-4^high ^Tck cells to extravasate endothelium in *ex vivo *cultures. Furthermore (and potentially of greater interest), we will determine the ability of CD4^+^CD45RO^+^VLA-4^high ^Tck cells to traffic to synovial grafts in the RA/severe combined immunodeficient mouse model, together with their potential to upregulate cytokines locally in this tissue.

The fact that the resting CD4^+^CD45RO^+ ^T cells from normal blood are unable to induce TNF-α from monocytes indicates that this effector function is acquired either due to *de novo *gene transcription and/or conformational change of a surface molecule during the differentiation process. Several cell surface molecules, including integrins and adhesion molecules, have previously been shown to be of importance in contact-dependent effector function, including CD69 [19], lymphocyte function-associated antigen-1 (CD11a and CD18) and others [18,36]. We confirmed that blockade of CD18 and CD69 on Tck cells reduced monocyte TNF-α production in the co-culture system, which is in agreement with previous studies [19,21,36]; also, in one single experiment blockade of CD49d (VLA-4) also reduced monocyte TNF-α production. More importantly, the role of these cell surface ligands was verified in the present study using the *ex vivo *RA synovial MNC culture system, reinforcing the relevance of Tck cells as surrogates for RA synovial T cells, and highlighting the potential role of these ligands in contributing to chronicity in RA. However, it is noteworthy that none of these ligation events were dominant in either system, and several different ligation events are potentially 'involved' in these contact-mediated events in inflammatory tissue, as was previously described [19].

VLA-4 also probably plays a role in trafficking of T cells to the synovial microenvironment (as discussed above). Interestingly, the chemokine receptor CXCR4 previously identified on RA synovial T cells [37] was also increased on day 8 Tck cells. CXCR4 plays a key role in trafficking T cells to inflamed synovium in response to ligands including stromal cell-derived factor-1 [37,38]. Therefore, antigen-independent activation by proinflammatory cytokines could not only induce cells capable of perpetuating disease by cellular contact with monocytes but also facilitate the accumulation of these pathogenic cells at the site of disease. We are currently exploring the role of VLA-4 (as discussed above), together with CXCR4 and stromal cell-derived factor-1, in migration of these Tck cells *in vivo *in the RA/SCID mouse model.

Although the series of cell sorting experiments indicated that enriching for CD4^+^CD45RO^+ ^T cells increased the Tck effector potency, variation between different experiments with different donors was very evident. One possible explanation is that regulatory T cells (or factors) are present in the cultures and suppress Tck effector function, either by modulating the generation of effector Tck cells and/or by modulating the ability of the monocytes to produce TNF-α after contact activation [39]. Consistent with this hypothesis was our observation that the proportion of CD4^+^CD25^+ ^cells increased in the Tck cultures (Table 1) and were found within the dividing population of CD4^+^CD45RO^+ ^cells (Figure 4a). We have preliminary data showing that CD25^+^Foxp3^+^CD127^- ^regulatory T cells are present in these Tck cultures and that they expand over the 8-day culture period at a faster rate than the Tck effector cells. Therefore, although the phenotype of the divided CD4^+^CD45RO^+ ^cells (Figure 4a) expressed more surface activation and adhesion molecules, including CD49d (Figure 4e), they may not necessarily be more potent effectors than the nondivided CD4^+^CD45RO^+ ^cells because CD25^+^Foxp3^+^CD127^- ^regulatory T cells are also present within these populations. We are currently determining in a separate and extensive study the modulatory effect of these CD25^+^Foxp3^+^CD127^- ^regulatory T cells upon the expansion of Tck cells, the acquisition of effector function and the direct effect on the monocytes in the co-culture model. In the RA synovial tissue we examined in this report (Figure 5), the proportion of CD4^+^CD25^+ ^cells was relatively low compared with that in other published reports. However, there is inconsistency in the published reports, with several describing an increase in the proportion of CD25^+^Foxp3^+^CD127^- ^regulatory T cells in the synovial fluid [40,41] and others describing a reduction in synovium compared with synovial fluid [42].

## Conclusion

The present study extends our previous observations on cytokine-activated T cells (Tck cells) and their potential in contributing to chronicity in inflammation. We found that CD4^+^CD45RO^+^CCR7^- ^effector memory T cells differentiate to an effector population upon cytokine stimulation *in vitro*, which mimics RA synovial T cells both in effector function and cell surface phenotype. By defining the phenotype of effector Tck cells, we have focused studies to gain further insight into the exact nature of contact-dependent activation of macrophages in RA. The cell surface molecules CD69, CD18 and CD49d were shown to plays roles in these contact-mediated events. The pathogenic potential of Tck cells is currently being explored further in the RA/severe combined immunodeficient model, which will also allow us to explore the modulatory effects of blocking candidate cell surface proteins on migratory cells and determine their effect on the pathogenesis of disease.

## Abbreviations

CCR = CC chemokine receptor; CFSE = carboxyfluoroscein succinimidyl ester; CXCR = CXC chemokine receptor; ELISA = enzyme-linked immunosorbent assay; HLA = human leucocyte antigen; IFN = interferon; IL = interleukin; MFI = mean fluorescence intensity; MNC = mononuclear cell; NK = natural killer; RA = rheumatoid arthritis; Tck cell = cytokine-activated T cell; TNF = tumour necrosis factor; Ttcr cell = T-cell receptor stimulated T cell; VCAM = vascular cell adhesion molecule; VLA = very late antigen.

## Competing interests

All authors concur with the submission and assert that the material submitted for publication has not previously been reported and is not under consideration for publication elsewhere.

## Authors' contributions

FMB initiated the study, reviewed analyzed data, created figures and wrote the manuscript. NMGS designed the study, acquired data, created figures and wrote the manuscript. SO designed the study, acquired the data, created figures and wrote the manuscript. CL acquired data, created figures and wrote the manuscript. PA acquired the data and created figures. PG acquired data. AA acquired data. ACP acquired data. PH acquired data. AF acquired data. JTB designed the study, analyzed the data, assembled and created figures, and wrote the manuscript. MF designed the study, acquired data and wrote the manuscript.
